# Dutch Perspectives Toward Governmental Trust, Vaccination, Myths, and Knowledge About Vaccines and COVID-19

**DOI:** 10.1001/jamanetworkopen.2021.40529

**Published:** 2021-12-30

**Authors:** Hamza Yousuf, Sander van der Linden, Ted van Essen, Diederik Gommers, Erik Scherder, Jagat Narula, Leonard Hofstra

**Affiliations:** 1 Department of Cardiology, Amsterdam University Medical Center, Vrije Universiteit (VU) University Medical Center, Amsterdam, the Netherlands; 2 Department of Psychology, School of Biology, University of Cambridge, Cambridge, United Kingdom; 3 Dutch Influenza Foundation, Amersfoort, the Netherlands; 4 Department of Intensive Care Medicine, Erasmus Medical Center, Rotterdam, the Netherlands; 5 Department of Clinical Neuropsychology, VU, Amsterdam, the Netherlands; 6 Mount Sinai Heart, Icahn School of Medicine at Mount Sinai, New York, New York

## Abstract

This survey study measures how opinions on trusting government and misconceptions about the COVID-19 vaccine are associated with vaccine hesitancy in the Netherlands.

## Introduction

As of November 24, 2021, 258 million confirmed COVID-19 cases had been reported worldwide, including 5.2 million COVID-19 related deaths.^1^ The race between vaccination and the occurrence of novel variants will ultimately determine the span of the pandemic. The World Health Organization (WHO) listed vaccine hesitancy as one of the top 10 global health threats. Politicization of acceptance of COVID-19 measures was illustrated by a study showing that US Democrats were more likely than Republicans to support regulation to contain SARS-CoV-2.^2^ European studies demonstrated decreased adherence to COVID-19 control measures in countries with low governmental trust.^3^ Herein, we report the results of a large nationwide survey study conducted in the Netherlands to investigate the association between governmental trust and vaccine confidence and misconceptions about both COVID-19 and vaccines.

## Methods

In December 2020, we distributed a survey through the largest Dutch national newspaper *De Telegraaf.* Participants completed a digital survey regarding (1) demographic information, (2) governmental trust on COVID-19 vaccination (adapted from a governmental trust survey during the H1N1 pandemic), (3) vaccine hesitancy, and (4) myths and knowledge about vaccines and COVID-19. Additional information on the formation of the digital survey is found in the eMethods in the Supplement. The responses to the survey were provided using either a 4-point Likert scale (strongly disagree, disagree, agree, or strongly agree) or a 5-point Likert scale (which adds an optional “I don’t know” response), unless indicated otherwise in the Table. Both strongly agree and agree are included in the agree designation. Likewise, strongly disagree and disagree are reported as disagree. “I don’t know” responses are not included in the reported responses. This study was reviewed and waived for official approval by the institutional review board of the Amsterdam UMC, Amsterdam, the Netherlands. Participants were required to provide informed consent, which was obtained digitally. Information on the background of participants, including race, ethnicity, educational level, income, and migrant background, was self-reported. This study followed the American Association for Public Opinion Research (AAPOR) reporting guideline.

**Table.** Survey Responses From Provaccination and Vaccine-Hesitant Groups

Survey question or response, %	Respondent group, %	
	Provaccination	Vaccine hesitant
**Governmental distrust** ^a^		
Agree		
How competent do you think the government is in dealing with COVID-19?	68.7	12.8
How honest do you think the government is with information regarding COVID-19?	76.5	9.4
To what extent do you believe that the actions of the government in response to COVID-19 are in your personal interest?	82.3	14.7
To what extent do you think the government will protect you against COVID-19?	77.7	11.1
**Vaccination motives and misinformation** ^b^		
Agree		
Because of my sense of responsibility toward society, I am getting vaccinated against COVID-19.	90.9	1.8
By being vaccinated against COVID-19, we can get back to the normal way of living as soon as possible (the situation before March 2020).	90.2	3.1
Because of the people in my own environment (eg, family, friends, or patients), I would get vaccinated against COVID-19.	94.4	5.6
Because of the benefits to my own health, I would get vaccinated against COVID-19.	88.1	2.4
I trust the advice of the government, research organizations, and manufacturers regarding the COVID-19 vaccine.	87.3	1.9
If you are vaccinated against COVID-19, you can still infect others with the virus.	19.8	4.8
Disagree		
There is still too little scientific knowledge about the safety of the COVID-19 vaccine.	60.1	2.3
I do not trust the safety of the COVID-19 vaccine. It is new and developed too quickly.	84.3	2.9
I think there is a lack of clarity about the rights and obligations with regard to vaccination.	53.3	8.7
I do not trust the pharmaceutical companies surrounding the COVID-19 vaccine.	81.8	5.6
I have no faith in politics surrounding the COVID-19 vaccine.	66.3	4.1
Because of information I read on the internet and social media, I would be less likely to be vaccinated against COVID-19.	88.9	38.4
I have already had COVID-19 and should be immune to it, so I am not taking a COVID-19 vaccine.	76.2	29.6
I want to wait and see before vaccinating myself with the COVID-19 vaccine, to see if there are any side effects that are not yet known.	83.9	28.2
For religious reasons, I will not get vaccinated against COVID-19.	97.7	74.1
In addition to effective ingredients in the COVID-19 vaccine, there are also ingredients that are harmful to me.	52.1	3.3
**Knowledge** ^b^		
Agree		
Patients with cardiovascular disease have a 5 times higher risk of dying from COVID-19.	64.3	48.4
People who are obese or overweight have higher risk of dying from COVID-19.	88.2	69.6
Patients with chronic lung disease have a higher risk of dying from COVID-19.	90.9	75.4
Patients with a higher age have a higher chance of dying from COVID-19.	91.3	78.3
Disagree		
Taking a COVID-19 vaccination actually leads to COVID-19.	89.3	28.5
Vaccination can lead to decline of strength of my immune system.	70.4	8.8
Vaccines can lead to the development of an autism spectrum disorder.	65.7	14.0
The COVID-19 vaccine (an mRNA vaccine) builds into your own DNA, and that is bad for your health.	66.9	8.9
**Vaccine hesitancy**		
Yes^c^		
Do you believe that vaccinations can protect you from serious diseases?	95.1	5.2
Do you think that most people like you have been vaccinated with all the recommended vaccinations, for example from the National Vaccination Program/hepatitis B?	76.5	62.9
Do leaders (religious or political leaders, teachers, health professionals) in your community support vaccinations?	46.7	31.4
No^d^		
Have you ever hesitated to get a vaccination, for example when traveling to a faraway country?	94.6	51.5
Have you ever refused a vaccination?	93.8	54.3
Are there any reasons you can think of why you should not get vaccinated?	75.2	3.1
Have you ever received or heard negative information about vaccinations?	36.9	8.7
Agree^b^		
Vaccinations are important to my health.	95.4	28.4
Vaccines are effective.	94.5	33.0
Getting vaccinated is important for the health of others in my community.	96.7	22.4
All vaccinations offered by the government program in my community are useful.	83.7	11.0
The information I receive about vaccines from the vaccination program is reliable.	75.5	11.7
Getting vaccines is a good way to protect myself from disease.	96.3	27.8
Disagree^b^		
New vaccines carry more risks than older vaccines.	47.6	8.7
Agree^a^		
In general, I do what my physician or health care professional recommends about vaccinations.	94.1	29.4
I am concerned about the possible adverse effects of vaccines.	75.9	6.0

^a^ Using a 4-point Likert scale including strongly disagree, disagree, agree, and strongly agree. Percentages of “agree” in the table include both strongly agree and agree responses. “Disagree” responses include both strongly disagree and disagree. In the Government distrust section, the 4-point Likert scale answer options to the open-ended questions were completely not, somewhat not, somewhat, and completely. Completely and somewhat were tabulated under agree, and somewhat not and completely not were tabulated under disagree.

^b^ Using a 5-point Likert scale including strongly disagree, disagree, agree, and strongly agree, as well as a choice of “I don’t know.” Percentages of “agree” in the table include both strongly agree and agree responses. “Disagree” responses include both strongly disagree and disagree. The “I don’t know” responses were not included in the results.

^c^ Using ordinal responses with 3 options: yes, no, and “I don’t know.” The “I don’t know” responses were not included in the results.

^d^ Using binary response: yes or no.

Descriptive analysis was used to present the outcome of the survey for the provaccination group and the vaccination-hesitant group. We used SPSS, version 27.0 for Mac (IBM) for statistical analysis.

## Results

A total of 24 722 participants completed the survey. The vaccine-hesitant group consisted of 12 640 participants (51.1%), indicating low acceptance for vaccination at the time of the survey (Table). We observed a higher incidence of vaccine hesitancy among respondents with a lower household annual income, migrant background, and lower educational level and among female respondents. The provaccination respondents showed higher confidence in information provided by the government (76.5% vs 9.4%) and were more likely to follow the advice of their physicians (94.1% vs 29.4%), compared with the vaccination-hesitant group. The acceptability of vaccination in the provaccination group was associated with a sense of responsibility toward society (90.9% vs 1.8%) and a heightened sense of protection of family and friends (94.4% vs 5.6%) compared with the vaccination-hesitant group. The vaccination-hesitant respondents were less likely to disagree with the statement “vaccinations could lead to an autism spectrum disorder (14.0% vs 65.7%). The vaccine-hesitant respondents were also less likely to disagree with the statement “The COVID-19 vaccine (an mRNA vaccine) builds into your own DNA, and that is bad for your health” (8.9% vs 66.9% and were less likely to disagree with the statement “Because of information I read on the internet and social media, I would be less likely to be vaccinated against COVID-19” (38.4% vs 88.9%), compared with the provaccination responders.

## Discussion

The findings of this survey study suggest substantial societal polarization surrounding vaccination, based predominantly on governmental distrust and belief in misinformation. Furthermore, societal and family responsibility was an important argument in the decision to get vaccinated among the provaccination respondents, but it was a negligible argument in the vaccination-hesitant group. Designation as Provaccination or Vaccination-Hesitant was self-reported. The question is how to successfully combat vaccination hesitancy. Previous studies showed that susceptibility to COVID-19 misinformation was associated with low trust in science and low numeracy skills, and they suggested public education as a solution.^4,5^ We recently demonstrated the effectiveness of debunking vaccination myths in a national campaign.^6^ In total, 18 280 people had died due to a COVID-19 infection as of November 19, 2021. The current 66.5% vaccination rate in the Netherlands compares favorably with the provaccination percentage presented herein. However, a recent surge in COVID-19 cases in areas with low vaccination rates highlights the importance of continuous campaigning for higher vaccination rates.

Major limitations of the study may be the insufficient representation of the Dutch population despite the large sample size and the time frame in which the study was performed, just before the start of the Dutch vaccination campaign. However, the data clearly show polarized views on governmental trust, trust in science, and the level of social responsibility between vaccination-hesitant respondents and provaccination respondents. We strongly recommend more intense public health education efforts to rebuild trust in the government, the scientific community, and the public health impact of vaccines.

## Notes

Supplement. **eMethods.** Supplemental Methods
eReferences
