# Supplementary material for: Dutch Perspectives Toward Governmental Trust, Vaccination, Myths, and Knowledge About Vaccines and COVID-19
Source: JAMA Netw Open. 2021 Dec 30;4(12):e2140529. doi: 10.1001/jamanetworkopen.2021.40529 (PMC8719233; doi:10.1001/jamanetworkopen.2021.40529)
Supplement: Supplement. — eMethods. Supplemental Methods eReferences [file jamanetwopen-e2140529-s001.pdf]

Supplemental Online Content

Yousuf H, van der Linden S, van Essen T, et al. Dutch perspectives toward governmental trust, vaccination, myths, and knowledge about vaccines and COVID-19. *JAMA Netw Open*. 2021;4(12):e2140529. doi:10.1001/jamanetworkopen.2021.40529

eMethods. Supplemental Methods

eReferences

This supplemental material has been provided by the authors to give readers additional information about their work.

© 2021 Yousuf H et al. *JAMA Network Open.*

eMethods. Supplemental Methods

Portions of the digital surveys were adapted from other surveys. The portion on governmental trust was adapted from a survey conducted during early stages of the H1N1 pandemic.[1] The portion on vaccine hesitancy was adapted from a World Health Organization working group vaccine hesitancy survey in Guatemala.[2]

eReferences

1. Freimuth VS, Musa D, Hilyard K, Quinn SC, Kim K. Trust during the early stages of the 2009 H1N1 pandemic. *J Health Commun*. 2014;19:321-339. https://doi.org/10.1080/10810730.2013.811323

2. Domek GJ, O'Leary ST, Bull S, et al. Measuring vaccine hesitancy: field testing the WHO SAGE working group on vaccine hesitancy survey tool in Guatemala. *Vaccine.* 2018;36:5273-5281. https://doi.org/10.1016/j.vaccine.2018.07.046

© 2021 Yousuf H et al. *JAMA Network Open.*
